# Germination and Early Growth of *Brassica juncea* in Copper Mine Tailings Amended with Technosol and Compost

**DOI:** 10.1155/2014/506392

**Published:** 2014-01-23

**Authors:** Luís A. B. Novo, Luís González

**Affiliations:** Department of Plant Biology and Soil Science, University of Vigo, As Lagoas, Marcosende, 36310 Vigo, Spain

## Abstract

Mine tailings represent a serious threat to the environment and human health; thus their restoration has become a major concern. In this study, the interactions between *Brassica juncea* and different mine soil treatments were evaluated in order to understand their effect on germination and early growth. Three soil treatments containing 25% and 50% of technosol and 30% of compost were prepared. Germination and early growth were assessed in soil and pore water extracts from the treatments. Unlike the untreated mine soil, the three treatments allowed germination and growth, achieving levels comparable to those of seedlings from the same species developed in normal conditions. The seedlings grown in 50% of technosol and 30% of compost exhibited greater germination percentages, higher growth, and more efficient mechanisms against oxidative stress, ascribed to the organic matter and nutrients content of these treatments. Considering the unequivocal ability of *B. juncea* for phytoremediation, the results suggest that technosol and compost may be an auspicious solution to allow the germination and early growth of this species in mine tailings.

## 1. Introduction

Mine soils are characterized by their instability, scarce cohesion, and low contents of nutrients and organic matter [1]. In addition, high levels of trace metals and the formation of acid drainage from sulphide oxidation with striking pH downfall contribute to the severe impairment of most metal mine tailings [2]. Restoration of the physical, chemical and biological properties of soils is essential to establish new vegetation [3]. The use of amendments is a common strategy to raise pH, stabilize trace metals, and favor revegetation of polluted soils [4–6].

Technosols are a new Reference Soil Group from the World Reference Base for Soil Resources [7] that contains a wide range of artifacts and materials of natural and anthropic origin. They include a variety of refuse-based soil-like landfills, cinders, sludge, or mine spoils whose properties and pedogenesis are dominated by their technical origin. When properly designed, they represent a cost-effective technology for soil remediation. Recently, a technosol built from sewage sludge, barley straw, and mussel shells has been tried on mine soil, yielding successful results on trace metals retention, pH, and soil organic matter increase [8].

Composts are a common tool for remediating metal contaminated soils [9, 10]. Modern management of waste has generated different types of composts, such as green compost from the agriculture sector, compost from household waste, or compost based on municipal sludge, among others [11]. Composts supply nutrients and organic matter and, equally important, bring beneficial microorganisms, sustaining their life in the soil [12]. Composts generally promote revegetation, which helps prevent the erosion of contaminated soils by wind and water [13]. In addition, cationic metals bind to exchange sites on the surface of organic matter, which reduces their mobility, avoiding metal leaching from soils [14].

Phytostabilization, a branch of phytoremediation, is an in situ process characterized by the use of soil amendments and plants tolerant to trace metals to promote ground revegetation, establishing a boundary that can reduce the migration of metals to air, surface, and ground water and, through the soil, decreasing its toxicity [15]. Phytoextraction is another subcategory of phytoremediation that can be defined as the ability to accumulate pollutants in harvestable plant parts [16]. Phytostabilization and phytoextraction constitute green and low cost techniques to mitigate the impact of trace metals in contaminated soils, capable of progressively overlapping more complex, expensive, and often harmful, chemical, and mechanical responses [15, 17, 18].

Combining the use of amendments such as technosol or compost and phytoremediation may constitute an effective solution to remediate polluted soils, whose success could exceed the results of each process alone. Plants require specific soil conditions, namely, pH, cation exchange capacity, and/or organic matter contents that limit their capacity to remediate the soils and the bioavailability of the contaminants [19]. These parameters can be satisfied with the appropriate choice of a tailor-made amendment, capable of providing the required characteristics to the polluted soils.

A vast number of species amongst the *Brassica* genus have been extensively tested for phytoremediation of trace metals. They have an intrinsic capacity not only to tolerate metals but also to promote their stabilization in the rhizosphere and accumulate them in high concentrations within different parts of the plant [20, 21]. *Brassica juncea* (L.) Czern and Coss is recurrently used in phytoremediation due to its aptitude to endure, stabilize, extract, and accumulate metals and its high biomass yield [22–26].

The aim of this study is to evaluate the interactions between *B. juncea* and the selected copper mine tailings treatments, in order to understand their effect on germination and early growth.

## 2. Material and Methods

### 2.1. Soil Sample Preparation and Experimental Design

Polluted soil (PS) was collected from a depleted copper mine located in Touro (NW Spain) (42°52′34′′N, 8°20′40′′W) and amended with technosol (TE) supplied by Tratamientos Ecológicos del Noroeste S.L. (Santiago de Compostela, A Coruña, Spain) and compost (CO) provided by Biocompost (Biocompost de Lugo, S.L., Cospeito, Lugo, Spain). The TE was prepared with mussel shells, sewage sludge, wood residue, and ashes in different proportions. Diverse organic wastes from the agriculture and food industries composed the CO. The physicochemical properties and selected metals concentrations in the technosol (TE) and compost (CO) can be found in a related study by Novo et al. [27].

The PS, exhibiting metal concentrations above the generic reference levels (GRL) for Galician soils on most metal species [28], was manually mixed and homogenized with TE and CO at proportions of (v/v): 25% TE (TE25), 50% TE (TE50), and 30% CO (CO30). These proportions were chosen upon a study by Novo et al. [6], in which similar amounts of technosol and compost were mixed with tailings from the same sampling site in the Touro mine, allowing the growth and development of *Salvia verbenaca*.

One week before starting the germination assays, “Rhizon” soil pore water samplers (Eijkelkamp Agrisearch Equipment, The Netherlands) were inserted into the soil of each treatment and PS. Vacuum tubes (10 mL) were attached through a Luer-lock system and hypodermic needles to extract pore water (PW). Rhizon samplers are an effective, simple, and inexpensive method for extracting soil solution that most likely represents the fraction of soil water taken up by plants [29]. Moreover, pore water obtained with Rhizon samplers is recommended for ecotoxicological tests [30].

Two different germination methods were carried out: direct soil germination on the three different treatments and PS, and germination on petri dishes containing PW extracts of PS, TE25, TE50, and CO30. Prior to germination tests through both methods, healthy *B. juncea* seeds acquired at Herbiseed (Herbiseed, Berkshire, United Kingdom) were selected according to uniform criteria and surface was sterilized in sodium hypochlorite 5% for 5 minutes.

Soil germination was monitored in trays with 5 × 5 alveoli (25 cm^2^ area). Each row was filled up with equal volumes of PS, TE25, TE50, and CO30. Seven seeds per alveolus of *B. juncea* were sowed and 5 replicates for each treatment were prepared. This process was carried out under greenhouse conditions: photoperiod of 10 : 14 h Light/Dark, temperature of 20 ± 2°C and 65 ± 5% relative air humidity. Soil humidity was kept constant with deionized water almost to field capacity, through gravimetric measurements performed on a daily basis.

For pore water germination, seeds were placed in sterilized Petri dishes (9 cm diameter) containing one layer of Whatman number 3 filter paper, at the rate of 15 seeds per dish. The filter papers were moistened with 5 mL of PW from the different treatments and PS. The Petri dishes, 5 replicates per treatment, were sealed with Parafilm in order to avoid evaporation and kept in a growth chamber at 24 ± 2°C with a 16 h photoperiod (130–135 *μ*mol m^−2^ s^−1^). Germination was considered when radicle was extended to at least one mm. The experiment was concluded after 8 days.

### 2.2. Soil and Pore Water Characterization

Polluted soil and the treatments were analyzed for pH according to Slattery et al. [31], total organic matter as per the method of Davies [32], and phosphorus (P_2_O_5_) by using inductively coupled plasma-optical emission spectrometry (ICP-OES; PerkinElmer Optima 4300 DV, PerkinElmer, Waltham, Massachusetts, USA). Total Kjeldahl-N (TN) was determined according to Bremner [33]. To determine total N content, an aliquot of each extract was analyzed by potentiometric titration with titrator equipment (Metrohm SM 702 Titrino).

Pore water samples were analyzed for pH with a pH meter, and dissolved organic carbon (DOC) using a TOC analyzer (Shimadzu TNM-1, Shimadzu, Tokyo, Japan).

In addition, trace metal contents (Cd, Cu, Pb, and Zn) in PW, PS, TE25, TE50, and CO30 were also measured by acid digestion using a mixture of HNO_3_ and HCl (1 : 3 v/v) in a microwave oven [34]. Analysis was processed by ICP-OES (PerkinElmer Optima 4300 DV, PerkinElmer, Waltham, Massachusetts, USA).

Table 1 summarizes some physicochemical characteristics and trace metal contents of PS, TE25, TE50, and CO30. Table 1 also shows the pH, DOC, and trace metal concentrations of the PW extractions for each treatment.

### 2.3. Seedlings Growth Analysis

Eight-day-old seedlings of each treatment were randomly picked from the soil and Petri dishes and divided in roots and shoots. Fresh biomass weight (FW) was determined immediately and dry biomass weight (DW) was assessed after oven drying for 48 h at 80°C and cooled down to room temperature. Additionally, the length of roots and shoots and leaf area were also determined.

The seed germination percentage was estimated through the following formula:

(1) $$\begin{array}{c} \text{germination}\;\left(\%\right)=100\times \frac{\text{number}\;\text{of}\;\text{germinated}\;\text{seeds}}{\text{total}\;\text{number}\;\text{of}\;\text{seeds}}\;. \end{array}$$

Seedling vigor index (SVI) was determined according to Abdul-Baki and Anderson [35]:

(2) $$\begin{array}{c} \text{SVI}=\text{germination}\;\left(\%\right)\times \text{root}\;\text{length}. \end{array}$$

### 2.4. Analytical Measurements

#### 2.4.1. Chlorophyll Content

Chlorophyll content was determined as described by Lichtenthaler [36]. Ten mg of fresh leaf samples was ground in 2 mL 80% (v/v) acetone. The homogenate was centrifuged at 3000 ×g for 10 min (Hettich Zentrifugen EBA 12R, A. Hettich, Tuttlingen, Germany). The absorbance of the supernatant was measured at 663.2 and 646.8, maximum absorbance of chlorophyll *a* and *b*, respectively (WPA Lightwave S2000, WPA, Cambridge, United Kingdom). Acetone 80% (v/v) was used as blank. Total chlorophyll content was estimated by totaling chlorophyll *a* and *b* contents. The results were expressed as mg g^−1^ leaf FW.

#### 2.4.2. Hydrogen Peroxide (H_2_O_2_)

The levels of H_2_O_2_ were estimated according to a modified version of the method by Alexieva et al. [37]. At the end of the experiment, 100 mg of fresh leaf samples was homogenized at 4°C with liquid nitrogen and 2 mL 0.1% (w/v) trichloroacetic acid (TCA). The homogenate was centrifuged at 12,000 ×g for 15 min and 0.5 mL of the supernatant was added to 0.5 mL 10 mmol potassium phosphate buffer (pH 7.0) and 1 mL 1 M potassium iodide (KI). Absorbance was read at 390 nm. For the blank, 0.5 mL of 0.1% (w/v) trichloroacetic acid (TCA), 0.5 mL of 10 mmol potassium phosphate buffer pH 7.0 and 1 mL of 1 M KI were used. The content of H_2_O_2_ was calculated by comparison with a standard calibration curve, plotted by using different known concentrations of H_2_O_2_.

#### 2.4.3. Lipid Peroxidation

The level of lipid peroxidation was assessed by a modification of the Hodges et al. [38] method, based on the measurement of the amount of malondialdehyde (MDA), which reacts with thiobarbituric acid (TBA) to form TBA-MDA complex.

100 mg fresh leaves samples were homogenized in 4 mL of 80% ethanol in a prechilled mortar and pestle at 4°C. The homogenate was then centrifuged at 3000 ×g for 5 min (Hettich Zentrifugen EBA 12R, A. Hettich, Tuttlingen, Germany). 1 mL of supernatant from crude extract was added to a test tube containing: (a) –TBA: 15 *μ*L 0.01% butylated hydroxytoluene + 985 *μ*L 20% TCA and (b) +TBA: 985 *μ*L 0.65% TBA + 15 *μ*L 0.01% butylated hydroxytoluene (w/v). Samples were then mixed vigorously, heated at 95°C for 30 min, and cooled on ice, before centrifugation at 3000 ×g for 10 min. Absorbance was read at 440 nm, 532 nm, and 600 nm by using UV-Visible Spectrophotometer (WPA Lightwave S2000, WPA, Cambridge, United Kingdom). Blanks were prepared according to the mixtures described previously, using 1 mL of 80% ethanol instead of supernatant. Total MDA equivalents were calculated according to the following equations:

(3) A=[(Abs 532+TBA)−(Abs 600+TBA)−(Abs 532−TBA−Abs 600−TBA)]B=[(Abs  440+TBA−Abs  600+TBA)×0.0571]MDA equivalents (nmol g−1 FW) =(A−B157)×103×(  solvent  volumesample  FW).

#### 2.4.4. Statistical Data

The Kolmogorov-Smirnov and Levene's tests were used to ensure the normality assumption and the homogeneity of variances, respectively. Analysis of variance (ANOVA) and homogeneity of variance tests were performed (*P* ≤ 0.05). Tukey posthoc comparisons between groups were carried out for homogeneous data, whereas Dunnett's T3 test was used in case of no homogeneity. For nonparametric data, the Kruskal-Wallis test and Mann-Whitney *U* test (*P* ≤ 0.05) were chosen. Pearson's correlation coefficients (*P* ≤ 0.01 and *P* ≤ 0.05) were used to determine the relationship between the values of different parameters. All statistical analyses were performed using IBM SPSS Statistics 19.0 (SPSS Inc., New York, USA).

## 3. Results

The three treatments exhibited a pronounced pH increment in comparison with PS (Table 1). The soil pH values among the treatments were significantly different (*P* ≤ 0.05), increasing 150.78%, 114.84%, and 104.79% in TE50, CO30, and TE25, respectively, in relation to PS. The pore water pH levels of TE50, CO30, and TE25 were increased by 119.19%, 93.94%, and 84.18%, respectively, although no significant differences were found between the three treatments. The contents of OM/DOC and nutrients in the compost treatment were significantly higher than in TE25 and TE50. The metal concentrations in TE25, TE50, and CO30 varied significantly according to the proportions of PS and technosol or compost in each treatment.

**Figure 1:**
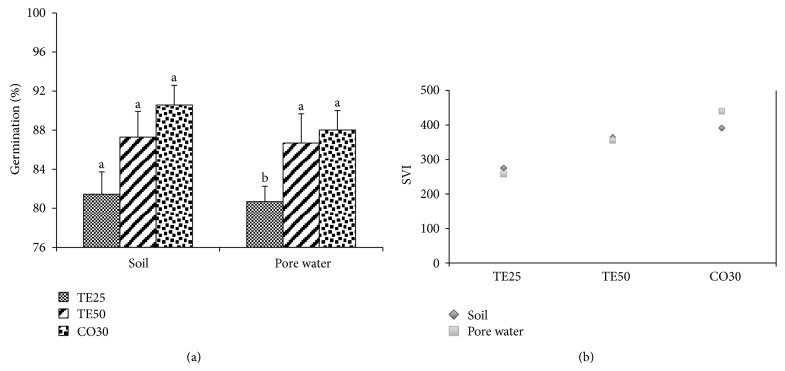
Germination percentage (a) and SVI (b) of *Brassica juncea* seedlings grown in soil and pore water of different copper mine tailings treatments. TE25 (25% of technosol), TE50 (50% of technosol), and CO30 (30% of compost). Data are means ± SE. Values followed by different letters differ significantly at *P* ≤ 0.05, *n* = 5.

No significant differences (*P* ≤ 0.05) were registered among the three treatments for the soil germination assays (Figure 1(a)). In PW, the germination percentages of *B. juncea* seeds sown in TE50 and CO30 were significantly higher than in TE25. Seeds did not germinate in PS through both methods. The SVI was markedly more elevated in TE50 and CO30, particularly in PW, wherein increments of 37.78% and 70.70%, respectively, were registered in contrast with TE25 (Figure 1(b)).

**Figure 2:**
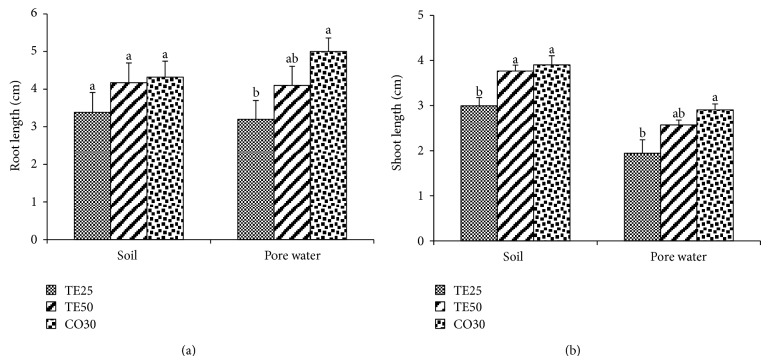
Length of roots (a) and shoots (b) of *Brassica juncea* seedlings grown in soil and pore water of different copper mine tailings treatments. TE25 (25% of technosol), TE50 (50% of technosol), and CO30 (30% of compost). Data are means ± SE. Values followed by different letters differ significantly at *P* ≤ 0.05, *n* = 5.

While no significant differences were observed in the root length measurements of the seedlings grown in soil, the radicle length values of CO30 in PW were considerably higher than those of TE25 (Figure 2(a)). The shoot length results of TE50 and CO30 in soil were significantly greater than those of TE25 by 25.71% and 30.22%, respectively (Figure 2(b)). In regard to PW, shoot length was maximal in CO30, denoting, however, significant differences solely in relation to TE25.

**Figure 3:**
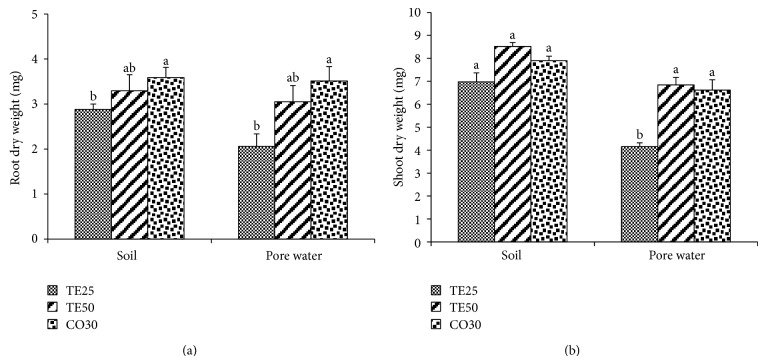
Dry weight of roots (a) and shoots (b) of *Brassica juncea* seedlings grown in soil and pore water of different copper mine tailings treatments. TE25 (25% of technosol), TE50 (50% of technosol), and CO30 (30% of compost). Data are means ± SE. Values followed by different letters differ significantly at *P* ≤ 0.05, *n* = 5.

The root dry weight results of *B. juncea* seedlings grown in soil and PW were analogous (Figure 3(a)). The dry weight of radicles developed in TE25 was significantly smaller than in CO30. Although no statistically meaningful differences were registered between TE50 and the other treatments, average increments of 14.35% and 48.10% in soil and PW, respectively, were registered in comparison with the root dry mass of TE25. The dry weight of the seedlings aerial parts was homogenous amongst the three treatments in soil assessments (Figure 3(b)). In PW, the shoot dry weight values were the highest in TE50 and CO30, exceeding TE25 by 64.74% and 59.18%, respectively.

**Figure 4:**
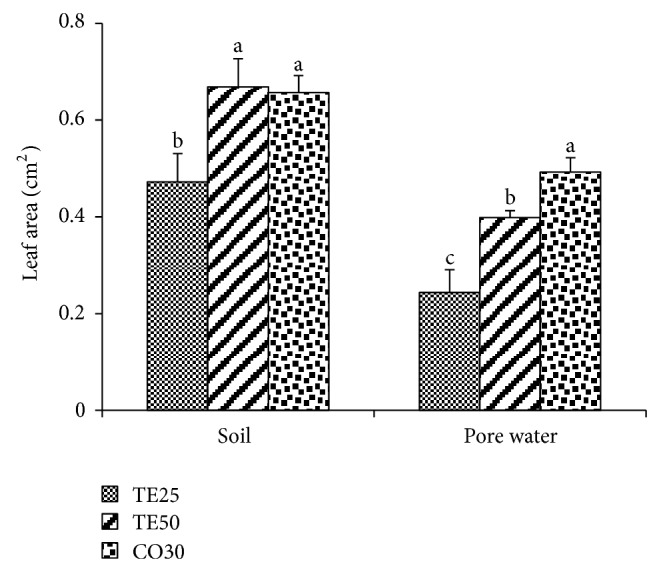
Leaf area of *Brassica juncea* seedlings grown in soil and pore water of different copper mine tailings treatments. TE25 (25% of technosol), TE50 (50% of technosol), CO30 (30% of compost). Data are means ± SE. Values followed by different letters differ significantly at *P* ≤ 0.05, *n* = 5.

Leaf area of seedlings grown in the soil of the TE50 and CO30 treatments significantly surpassed TE25 by 41.61% and 39.15%, respectively (Figure 4). In PW, significant differences were found among the three treatments, with CO30 marking the largest leaf area, followed by TE50 and then by TE25.

The concentrations of hydrogen peroxide were found higher in the seedlings developed in the soil of TE25 (Table 2). In detail, the results of TE25 were 4.43% higher than TE50 and significantly greater by 9.26% than CO30. In the PW assay, the levels of H_2_O_2_ were significantly different among the three treatments. The seedlings of TE25 displayed the highest results, outdoing TE50 and CO30 by 11.04% and 37.33%, respectively.

In soil, lipid peroxidation was significantly higher in TE25 (Table 2). The MDA concentrations of this treatment were 19.68% and 22.57% more elevated than in TE50 and CO30, respectively. With respect to PW, the three treatments were significantly different between each other. Again, the seedlings of TE25 had an excess of 10.75% and 23.90% in MDA content, relative to that in TE50 and CO30, correspondingly.

Both assays presented significant alterations in chlorophyll content among all the treatments (Table 2). In soil, the amount of chlorophyll in CO30 was 17.78% and 39.47% greater than TE50 and TE25, respectively. Similarly, the *B*.* juncea* seedlings developed in the PW extracted from CO30, suffered chlorophyll increments of 24.80% relative to TE50 and 99.44% with respect to TE25.

Correlation coefficients of 0.888 and 0.921 were found between root length, and the organic matter and nitrogen content of soil, respectively (Table 3). A correlation coefficient of 0.766 (significant at the 0.05 level, two tailed) was obtained between root length and the levels of dissolved organic carbon in pore water.

## 4. Discussion

Seed germination is one of the most critical stages in the life cycle of a plant, as it represents the first exchange interface with the surrounding medium [39]. Mine soils comprise a wide range of limiting factors for plant growth, such as extremely acidic pH and low content of nutrients [1, 40, 41]. The absence of germination in PS, both in soil and pore water, was very likely related to the elevated acidity of the mine tailings. The addition of technosol and compost increased pH, allowing germination and seedling growth. Although no statistically significant differences were found in the soil assay, the percentages of both germination methods suggest that the pH ranges generated by TE50 and CO30 allowed the achievement of better germination results.

Roots are generally more affected by trace metals toxicity than shoots, because roots are responsible for their absorption and primary accumulation [42, 43]. The mine tailings used in this study contained a cocktail of trace metals, whose individual and combined deleterious effects on plants frequently include inhibition of root elongation, seedling growth, and leaf expansion [44, 45]. In general, the amendments secured root and shoot growth results comparable to those of seedlings of the same species developed without the effect of metal toxicity [46]. In consequence the SVI of the seedlings subjected to the three treatments presented promising results in regard to upcoming plant growth phases. Nonetheless, significant differences found in the length and dry weight of root and shoot, as well as leaf area, through both germination methods, evoke the superiority of TE50 and CO30 in the promotion of seedling growth. Mine soils are known to have a scarcity of nutrients that seriously compromises plant growth; however the application of technosol and compost is an effective solution to not only raise pH and stabilize trace metals but also provide nutrients and organic matter that are fundamental for normal plant development [1, 5, 6]. The correlations verified between root length and the organic matter and nitrogen content of soil (Table 3), and between root length and the levels of dissolved organic carbon in pore water, indicate the importance of the amendments for plant growth and explain the advantage of TE50 and CO30 over TE25.

Trace metals are known to disrupt redox homeostasis by promoting the formation of free radicals and reactive oxygen species (ROS) like singlet oxygen, superoxide radicals, hydrogen peroxide, and hydroxyl radicals [47]. The coordinated counteraction of antioxidant enzymes such as superoxide dismutase and ascorbate peroxidase has been proposed to explain the mechanism used by plants to prevent the production of hydroxyl radicals and scavenge ROS [48, 49]. The assessments to the contents of hydrogen peroxide and MDA (a cytotoxic product of lipid peroxidation and an indicator of free radical synthesis) revealed less oxidative stress in the seedlings grown in TE50 and CO30. This may be ascribed to the superior antioxidative response of these seedlings as result of the impact of their respective treatments in the soil quality and its subsequent effect on the ecophysiology and biochemistry of the plants.

In addition, trace metals stress usually reduces chlorophyll synthesis due to the inhibition of enzymes involved in its synthetic pathway [44, 47]. The seedlings of *B. juncea* grown in TE50 and CO30, especially the latter, denoted higher chlorophyll content and thus greater fitness against metal toxicity.

Further research about the effect of the treatments on additional ecophysiological and biochemical traits would be important to complement the results of this study. Moreover, the evaluation of the plant development in upcoming growth stages is crucial to determine the suitability of *B. juncea* for phytoremediation of mine tailings treated with compost and technosol. Nevertheless, the results are auspicious given that germination and early growth constitute critical steps in the plant life cycle.

## 5. Conclusion

The results clearly expressed the ability of *B. juncea* to germinate and grow in the three copper mine tailings treatments, achieving growth levels within the spectrum of seedlings from the same species developed without the detrimental effects of metal toxicity. Nevertheless, unambiguous differences were found between the treatments concerning plant growth and the amount of stress signalers. The seedlings developed in TE50 and CO30, particularly in the latter, displayed an overall superiority over TE25 through both germination methods, achieving higher germination percentages, greater growth, and more efficient mechanisms against oxidative stress. Technosol and compost proved essential to amend the mine tailings, raising the pH and providing nutrients indispensable for the germination and early growth of *B. juncea*.

In conclusion, the results of this study are promising considering that germination and early growth represent crucial phases of the plant life cycle. Still, additional research to the plant development in further growth stages is fundamental to determine the fitness of *B. juncea* for phytoremediation of mine tailings treated with compost and technosol.

## Figures and Tables

**Table 1:** Physicochemical properties and selected trace metals concentrations in soil and pore water of the polluted soil (PS) and the three treatments: polluted soil amended with 25% of technosol (TE25), polluted soil amended with 50% of technosol (TE50), and polluted soil amended with 30% of compost (CO30).

Soil				
	PS	TE25	TE50	CO30
pH∗	2.56 ± 0.09	5.24 ± 0.20^c^	6.42 ± 0.18^a^	5.50 ± 0.20^b^
OM (%)∗	0.03 ± 0.00	5.45 ± 0.17^c^	10.66 ± 0.29^b^	18.85 ± 0.61^a^
TN (%)∗	0.11 ± 0.01	0.47 ± 0.02^c^	0.79 ± 0.03^b^	0.97 ± 0.03^a^
P_2_O_5_ (mg kg^−1^)∗	197.5 ± 6.45	281.51 ± 9.44^c^	368.25 ± 12.02^b^	8496.47 ± 269.78^a^

Cd (mg kg^−1^)∗	0.41 ± 0.02	0.40 ± 0.02^a^	0.38 ± 0.02^b^	0.39 ± 0.01^b^
Cu (mg kg^−1^)∗	260.45 ± 7.50	209.14 ± 7.29^a^	151.91 ± 6.24^c^	198.01 ± 6.29^b^
Pb (mg kg^−1^)	8.59 ± 0.72	17.43 ± 1.39^b^	27.28 ± 1.77^a^	17.38 ± 1.38^b^
Zn (mg kg^−1^)∗	53 ± 1.58	60.80 ± 1.67^c^	67.15 ± 1.82^b^	70.72 ± 1.11^a^

Pore Water				
pH	2.97 ± 0.32	5.47 ± 0.68^a^	6.51 ± 0.37^a^	5.76 ± 0.54^a^
DOC (%)	n.d.	1.32 ± 0.14^c^	2.78 ± 0.21^b^	3.57 ± 0.17^a^

Cd (mg kg^−1^)∗	n.d.	n.d.	n.d.	n.d.
Cu (mg kg^−1^)∗	21.84 ± 1.72	13.9 ± 1.18^a^	16.19 ± 1.38^a^	14.73 ± 1.07^a^
Pb (mg kg^−1^)	1.21 ± 0.10	0.27 ± 0.02^b^	0.98 ± 0.07^a^	1.18 ± 0.08^a^
Zn (mg kg^−1^)∗	0.34 ± 0.01	0.32 ± 0.01^b^	0.35 ± 0.01^b^	0.47 ± 0.02^a^

Each value is the mean of five replicate measurements ± SE (standard error). n.d.: not detected. Different letters indicate significant difference between treatments at *P* ≤ 0.05.

∗Data from Novo et al. [6].

**Table 2:** Hydrogen peroxide, MDA, and chlorophyll contents in seedlings of *Brassica juncea* grown in soil and pore water of the three treatments: polluted soil amended with 25% of technosol (TE25), polluted soil amended with 50% of technosol (TE50), and polluted soil amended with 30% of compost (CO30).

Soil			
	TE25	TE50	CO30
H_2_O_2_ (*μ*mol g^−1^ FW)	4.01 ± 0.02^a^	3.84 ± 0.11^ab^	3.67 ± 0.07^b^
MDA (nmol g^−1^ FW)	43.17 ± 1.11^a^	36.07 ± 0.96^b^	35.22 ± 1.47^b^
Chl (mg g^−1^ FW)	1.14 ± 0.02^c^	1.35 ± 0.03^b^	1.59 ± 0.07^a^

Pore Water			
H_2_O_2_ (*μ*mol g^−1^ FW)	4.93 ± 0.12^a^	4.44 ± 0.07^b^	3.59 ± 0.05^c^
MDA (nmol g^−1^ FW)	44.90 ± 0.68^a^	40.54 ± 0.81^b^	36.24 ± 0.55^c^
Chl (mg g^−1^ FW)	0.84 ± 0.02^c^	1.34 ± 0.04^b^	1.67 ± 0.07^a^

Each value is the mean of five replicate measurements ± SE. Different letters indicate significant difference between treatments at *P* ≤ 0.05.

**Table 3:** Pearson correlation analysis between root length and the physicochemical properties of the soil treatments.

Harvestable amount	pH	OM	TN	P_2_O_5_
Root length	0.452	0.888∗∗	0.921∗∗	0.630

^**^Correlation is significant at the 0.01 level (two tailed).

## References

[1] Asensio V., Vega F. A., Andrade M. L., Covelo E. F. (2013). Technosols made of wastes to improve physico-chemical characteristics of a copper mine soil. *Pedosphere*.

[2] Alcolea A., Vázquez M., Caparrós A., Ibarra I., García C., Linares R., Rodríguez R. (2012). Heavy metal removal of intermittent acid mine drainage with an open limestone channel. *Minerals Engineering*.

[3] Doran J., Jones A., Arshad M., Gilley J. (1999). *Determinants of Soil Quality and Health*.

[4] Faz A., Carmona D. M., Zanuzzi A., Mermut A. R. (2008). Pig manure application for remediation of mine soils in Murcia Province, SE Spain. *TheScientificWorldJOURNAL*.

[5] Melgar-Ramírez R., González V., Sánchez J. A., García I. (2012). Effects of application of organic and inorganic wastes for restoration of sulphur-mine soil. *Water, Air, & Soil Pollution*.

[6] Novo L. A. B., Covelo E. F., González L. (2013). The potential of *Salvia verbenaca* for phytoremediation of copper mine tailings amended with technosol and compost. *Water, Air, & Soil Pollution*.

[7] FAO (2006). *World Reference Base For Soil Resources*.

[8] Vega F. A., Covelo E. F., Andrade M. L. (2009). Effects of sewage sludge and barley straw treatment on the sorption and retention of Cu, Cd and Pb by coppermine Anthropic Regosols. *Journal of Hazardous Materials*.

[9] Businelli D., Massaccesi L., Said-Pullicino D., Gigliotti G. (2009). Long-term distribution, mobility and plant availability of compost-derived heavy metals in a landfill covering soil. *Science of the Total Environment*.

[10] Farrag K., Senesi N., Nigro F., Petrozza A., Palma A., Shaarawi S., Brunetti G. (2012). Growth responses of crop and weed species to heavy metals in pot and field experiments. *Environmental Science and Pollution Research*.

[11] Sæbø A., Ferrini F. (2006). The use of compost in urban green areas—a review for practical application. *Urban Forestry & Urban Greening*.

[12] Amlinger F., Götz B., Dreher P., Geszti J., Weissteiner C. (2003). Nitrogen in biowaste and yard waste compost: dynamics of mobilisation and availability—a review. *European Journal of Soil Biology*.

[13] Pedrol N., Puig C. G., Souza P., Forján R., Vega F. A., Asensio V., González L., Cerqueira B., Covelo E. F., Andrade L. (2010). Soil fertility and spontaneous revegetation in lignite spoil banks under different amendments. *Soil and Tillage Research*.

[14] Soler-Rovira P., Madejón E., Madejón P., Plaza C. (2010). In situ remediation of metal-contaminated soils with organic amendments: role of humic acids in copper bioavailability. *Chemosphere*.

[15] Mendez M. O., Maier R. M. (2008). Phytostabilization of mine tailings in arid and semiarid environments—an emerging remediation technology. *Environmental Health Perspectives*.

[16] Chaney R. L., Malik M., Li Y. M., Brown S. L., Brewer E. P., Angle J. S., Baker A. J. M. (1997). Phytoremediation of soil metals. *Current Opinion in Biotechnology*.

[17] Cheng S. (2003). Heavy metals in plants and phytoremediation. *Environmental Science and Pollution Research*.

[18] Conesa H. M., Evangelou M. W. H., Robinson B. H., Schulin R. (2012). A critical view of current state of phytotechnologies to remediate soils: still a promising tool?. *The Scientific World Journal*.

[19] Kayser A., Wenger K., Keller A., Attinger W., Felix H. R., Gupta S. K., Schulin R. (2000). Enhancement of phytoextraction of Zn, Cd, and Cu from calcareous soil: the use of NTA and sulfur amendments. *Environmental Science & Technology*.

[20] Nanda Kumar P. B. A., Dushenkov V., Motto H., Raskin I. (1995). Phytoextraction: the use of plants to remove heavy metals from soils. *Environmental Science & Technology*.

[21] Ebbs S. D., Kochian L. V. (1997). Toxicity of zinc and copper to *Brassica* species: implications for phytoremediation. *Journal of Environmental Quality*.

[22] Prasad M. N. V., de Oliveira Freitas H. M. (2003). Metal hyperaccumulation in plants—biodiversity prospecting forphytoremediation technology. *Electronic Journal of Biotechnology*.

[23] Bluskov S., Arocena J. M., Omotoso O. O., Young J. P. (2005). Uptake, distribution, and speciation of chromium in *Brassica juncea*. *International Journal of Phytoremediation*.

[24] Meyers D. E. R., Auchterlonie G. J., Webb R. I., Wood B. (2008). Uptake and localisation of lead in the root system of *Brassica juncea*. *Environmental Pollution*.

[25] Purakayastha T. J., Viswanath T., Bhadraray S., Chhonkar P. K., Adhikari P. P., Suribabu K. (2008). Phytoextraction of zinc, copper, nickel and lead from a contaminated soil by different species of *Brassica*. *International Journal of Phytoremediation*.

[26] Dede G., Ozdemir S., Dede O. H. (2012). Effect of soil amendments on phytoextraction potential of *Brassica juncea* growing on sewage sludge. *International Journal of Environmental Science & Technology*.

[27] Novo L. A. B., Covelo E. F., González L. (2013). The use of waste-derived amendments to promote the growth of Indian mustard in copper mine tailings. *Minerals Engineering*.

[28] Macías F. V., Calvo R. A. (2009). *Generic Reference Levels of Heavy Metals and Other Trace Elements in Soils of Galicia*.

[29] Clemente R., Dickinson N. M., Lepp N. W. (2008). Mobility of metals and metalloids in a multi-element contaminated soil 20 years after cessation of the pollution source activity. *Environmental Pollution*.

[30] Tiensing T., Preston S., Strachan N., Paton G. I. (2001). Soil solution extraction techniques for microbial ecotoxicity testing: a comparative evaluation. *Journal of Environmental Monitoring*.

[31] Slattery W., Conyers M., Aitken R., Peverill K., Sparrow L., Reuter D. (1999). Soil pH, aluminium, manganese and lime requirement. *Soil Analysis: An Interpretation Manual*.

[32] Davies B. E. (1974). Loss-on-ignition as an estimate of soil organic matter. *Soil Science Society of America Journal*.

[33] Bremner J. M., Sparks D. L. (1996). Nitrogen-total. *Methods of Soil Analysis*.

[34] Moor C., Lymberopoulou T., Dietrich V. J. (2001). Determination of heavy metals in soils, sediments and geological materials by ICP-AES and ICP-MS. *Mikrochimica Acta*.

[35] Abdul-Baki A. A., Anderson J. D. (1973). Relationship between decarboxylation of glutamic acid and vigor in soybean seed. *Crop Science*.

[36] Lichtenthaler H. K. (1987). Chlorophylls and carotenoids: pigments of photosynthetic biomembranes. *Methods in Enzymology*.

[37] Alexieva V., Sergiev I., Mapelli S., Karanov E. (2001). The effect of drought and ultraviolet radiation on growth and stress markers in pea and wheat. *Plant, Cell and Environment*.

[38] Hodges D. M., DeLong J. M., Forney C. F., Prange R. K. (1999). Improving the thiobarbituric acid-reactive-substances assay for estimating lipid peroxidation in plant tissues containing anthocyanin and other interfering compounds. *Planta*.

[39] Solanki R., Dhankhar R. (2011). Biochemical changes and adaptive strategies of plants under heavy metal stress. *Biologia*.

[40] Aguilar J., Dorronsoro C., Fernández E., Fernández J., García I., Martín F., Simón M. (2004). Soil pollution by a pyrite mine spill in Spain: evolution in time. *Environmental Pollution*.

[41] Shi X., Zhang X., Chen G., Chen Y., Wang L., Shan X. (2011). Seedling growth and metal accumulation of selected woody species in copper and lead/zinc mine tailings. *Journal of Environmental Sciences*.

[42] Marques M. O., Melo W. J., Marques T. A., Tsutiya M. T. (2002). Heavy metals and the use of biosolids in agriculture. *Biosolids in Agriculture*.

[43] Öncel I., Keles Y., Üstüun A. S. (2000). Interactive effects of temperature and heavy metal stress on the growth and some biochemical compounds in wheat seedlings. *Environmental Pollution*.

[44] Nagajyoti P. C., Lee K. D., Sreekanth T. V. M. (2010). Heavy metals, occurrence and toxicity for plants: a review. *Environmental Chemistry Letters*.

[45] Sherameti I., Varma A. (2010). *Soil Heavy Metals*.

[46] Bauddh K., Singh R. P. (2011). Differential toxicity of cadmium to mustard (*Brassica juncia* L.) genotypes under higher metal levels. *Journal of Environmental Biology*.

[47] Hossain M. A., Piyatida P., da Silva J. A. T., Fujita M. (2012). Molecular mechanism of heavy metal toxicity and tolerance in plants: central role of glutathione in detoxification of reactive oxygen species and methylglyoxal and in heavy metal chelation. *Journal of Botany*.

[48] Ashraf M., Harris P. J. C. (2004). Potential biochemical indicators of salinity tolerance in plants. *Plant Science*.

[49] Gill S. S., Tuteja N. (2010). Reactive oxygen species and antioxidant machinery in abiotic stress tolerance in crop plants. *Plant Physiology and Biochemistry*.

